# Short-term comparison of preoperative and postoperative pain after indirect decompression surgery and direct decompression surgery in patients with degenerative spondylolisthesis

**DOI:** 10.1038/s41598-020-76028-y

**Published:** 2020-11-03

**Authors:** Akihiko Hiyama, Hiroyuki Katoh, Daisuke Sakai, Masahiro Tanaka, Masato Sato, Masahiko Watanabe

**Affiliations:** Department of Orthopaedic Surgery, Tokai University School of Medicine, 143 Shimokasuya, Isehara, Kanagawa 259-1193 Japan

**Keywords:** Spine regulation and structure, Neurological disorders, Neuroscience, Neurology

## Abstract

The purpose of this study was to compare the short-term clinical outcomes between extreme lateral interbody fusion (XLIF) and minimally invasive surgery (MIS)–transforaminal interbody fusion (TLIF) in patients with degenerative spondylolisthesis with stenosis. One hundred-six patients were enrolled; 44 were treated with MIS–TLIF (direct decompression group; DP), and 62 were treated with XLIF (indirect decompression group; IDP). Perioperative indexes included operation time and intraoperative bleeding. Perioperative indexes preoperative and postoperative numeric rating scale (NRS) scores for low back pain (NRS-BP), leg pain (NRS-LP), and leg numbness (NRS-LN), and the preoperative score on the Japanese version of the painDETECT questionnaire (PDQ-J) were also assessed. The average follow-up period for the collection of NRS scores was 12.6 months. The operation time was significantly shorter in the IDP than in the DP group (109.9 ± 35.4 vs. 153.3 ± 50.9 min; *p* < 0.001). Intraoperative blood loss was also significantly less in the IDP group than in the DP group (85.4 ± 125.4 vs. 258.3 ± 220.4 mL; *p* < 0.001). The PDQ-J score and preoperative NRS scores (NRS-BP, NRS-LP, and NRS-LN) did not differ significantly between groups. Less improvement in the NRS-BP (ΔNRS-BP) was observed in the DP group than in the IDP group (*p* < 0.05). Although pain improved after surgery in both groups, IDP surgery was advantageous in minimizing bleeding and preserving posterior support elements such as the facet joints, lamina, and paraspinal muscles. These findings suggest that this may have contributed to the higher rate of improvement in low back pain compared with DP surgery.

## Introduction

Ozgur et al. published the first article in 2006 about surgery using the lateral transpsoas approach for interbody fusion^1^. Since then, this technique has become a common approach to achieving interbody fusion^2,3^. Moreover, lateral lumbar interbody fusion (LLIF) is recognized as a minimally invasive surgical technique that allows access to the intervertebral disc space and vertebral bodies via a retroperitoneal transpsoas approach.

**Figure 1:**
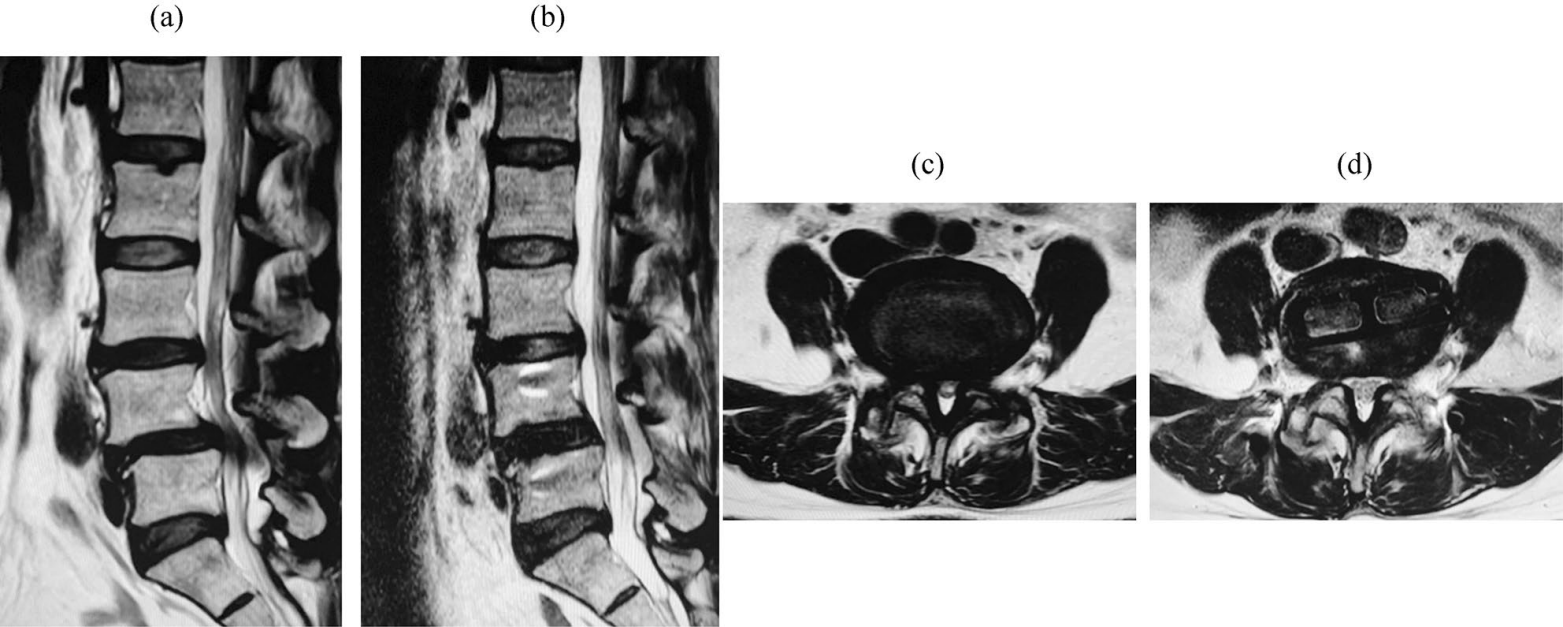
Images of a 54-year-old female patient with L4 spondylolisthesis. (**a**) Preoperative sagittal MRI image. (**b**) Postoperative sagittal MRI image. (**c**) Preoperative axial MRI image at the L4–5 disc level. (**d**) Postoperative axial MRI image at the L4–5 disc level 12 months after XLIF with PPS fixation (IDP) surgery. Pain improved from preoperative to postoperative measurements: back pain, NRS-BP from 9 to 0; leg pain, NRS-LP from 0.5 to 0; and leg numbness, NRS-LN from 9 to 0. MRI, magnetic resonance imaging; IDP, indirect decompression; NRS, numeric rating scale; NRS-BP, NRS for low back pain; NRS-LP, NRS for leg pain; NRS-LN, NRS for leg numbness.

Although good clinical outcomes have been reported for traditional posterior lumbar interbody (PLIF) and transforaminal interbody fusion (TLIF), complications such as incidental durotomy, nerve injury, massive blood loss, and epidural hematoma have also been documented^4–6^. In contrast to PLIF and TLIF, which often rely on direct decompression (DP) of neural elements such as removal of the ligamentum flavum, laminectomy, or facetectomy, LLIF can provide indirect decompression (IDP) when the large interbody cage ligament taxis technique is used^7^ (Fig. 1). The greatest advantage of LLIF is that it can indirectly affect decompression of spinal stenosis without destroying posterior elements. The benefits of this procedure over conventional open surgery are also reduced blood loss, shorter hospital stays, and reduced postoperative pain.

Previous studies of LLIF have shown significant radiographic improvements^8,9^. Patients with symptomatic lumbar stenosis exhibit increased foraminal area, foraminal height, disc height, and central canal diameter after LLIF treatment. In addition, statistically, significant improvements have been reported on a visual analogue scale (VAS) scores for pain and quality of life after minimally invasive LLIF treatment^10^. However, few studies have compared the clinical outcomes in patients treated with the IDP and DP techniques. The purpose of this study was to compare the clinical outcomes, such as the changes in low back pain, leg pain, and leg numbness, between patients receiving IDP surgery using LLIF with extreme lateral interbody fusion (XLIF, NuVasive, Inc., CA, USA) and those receiving DP surgery using minimally invasive surgery (MIS) involving TLIF (MIS–TLIF).

## Results

Retrospective data were collected and analyzed for 106 patients who met the inclusion/exclusion criteria. The average follow-up period for collecting NRS scores was 12.6 ± 5.6 months (median; 12.0 months). The average age at the time of surgery was 68.8 years, and 60.4% of patients were men. Information about the two groups of patients is presented in Table 1. Patients were matched for comorbidities between two groups.

**Table 1:** Comparison of two groups.

Characteristic	ALL	DP group	IDP group	*p* value
**No. of patients**	106	44	62 (S32 D30)	−
Age (years)	68.8 ± 9.2	66.9 ± 10.3	70.2 ± 8.1	0.104
Sex (M, F)	64: 42	27: 17	37: 25	0.862
**No. of fusion levels**	1.3 ± 0.6	1.3 ± 0.4	1.4 ± 0.6	0.394
One-level	76	33	43	
Two-levels	25	11	14	
Three-levels	5	0	5	
Four-levels	0	0	0	
**No. of fusion spine levels**	141	55	86	
L1–2	2	0	2	
L2–3	12	0	12	
L3–4	44	13	31	
L4–5	70	29	41	
L5–S1	13	13	0	
Blood loss (ml)	157.1 ± 191.1	258.3 ± 220.4	85.4 ± 125.4 S: 57.2 ± 62.3 D: 115.5 ± 166.4	< 0.001
Time in operating room (min)	127.9 + 47.6	153.3 + 50.9	109.9 + 35.4 S: 92.8 ± 26.7 D: 128.0 ± 35.5	< 0.001
Preoperative Hb (g/dl)	13.7 ± 1.7	13.8 ± 1.8	13.6 ± 1.7	0.557
Postoperative Hb day 1 (g/dl)	11.7 ± 1.7	11.4 ± 1.6	11.9 ± 1.8	0.151
Change in Hb preoperative to postoperative day 1 (g/dl)	− 2.0 ± 1.1	− 2.4 ± 1.1	− 1.7 ± 1.0	< 0.01

All values are in the mean ± standard deviation.

DP, direct decompression; IDP, indirect decompression; S, single position; D, dual position; Hb, hemoglobin.

The percentages of patients who underwent MIS–TLIF were 41.5% (44 in the DP group) and 58.5% (62 in the IDP group) with XLIF and PPS fixation. We found no significant differences between groups for the mean age (66.9 ± 10.3 vs. 70.2 ± 8.1 years), sex (male/female ratios, 27:17 vs. 37:25), or the number of fused spine levels. The procedure involved one level in 33 patients and two levels in 11 patients in the DP group. In the IDP group, the procedure involved one level in 43 patients, two levels in 14 patients, and three levels in 5 patients; no patient underwent a procedure involving more than three levels. The operation time was significantly shorter in the IDP group than in the DP group (109.9 ± 35.4 vs. 153.3 ± 50.9 min; *p* < 0.001). The operation times differed significantly between the single and dual positions: 92.8 and 128.0 min, respectively (*p* < 0.001).

The intraoperative blood loss was significantly less in the IDP group than in the DP group (85.4 ± 125.4 vs. 258.3 ± 220.4 mL; *p* < 0.001). The preoperative and postoperative hemoglobin (Hb) levels did not differ significantly, but the change in Hb level was significantly smaller in the IDP group than in the DP group.

Among all patients with low back pain and/or leg pain, 54 (50.9%) had nociceptive pain, 17 (16.0%) had neuropathic pain, and 35 (33.0%) had an unclear type of pain (mixed pain) at their preoperative examination (Table 2). The PDQ-J score did not differ significantly between the two groups (*p* = 0.574).

**Table 2:** Comparison of two groups for the preoperative PDQ-J score.

Characteristic	ALL	DP group	IDP group	*p* value
No. of patients	106	44	62	
**PDQ-J**				
Average score	12.6 + 5.6	12.2 + 5.6	12.9 + 5.8	0.574
0–12 (NocP)	54	23	31	
13–18 (Unclear)	35	14	21	
19–38 (NeP)	17	7	10	

PDQ-J, Japanese version of the painDETECT questionnaire; NocP, Nociceptive pain; NeP, Neuropathic pain.

**Figure 2:**
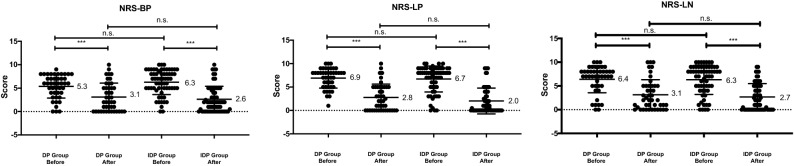
Graphs showing the preoperative and postoperative NRS scores in the two groups. NRS, numeric rating scale; NRS-BP, NRS for low back pain; NRS-LP, NRS for leg pain; NRS-LN, NRS for leg numbness. n.s., not significant; *p < 0.05, ***p < 0.001 indicates significant differences between groups.

Preoperatively, all patients had low back pain (mean NRS-BP of 5.9 ± 2.6), leg pain (mean NRS-LB of 6.8 ± 2.5), or leg numbness (mean NRS-LN of 6.3 ± 3.0), but the scores for these components did not differ significantly between the two groups. The postoperative NRS-BP scores at the final follow-up were 3.1 ± 3.0 and 2.6 ± 2.8 for the DP and IDP groups, respectively, but did not differ significantly (*p* = 0.940). A similar tendency was observed for the NRS-LN and NRS-LP scores (Fig. 2).

**Figure 3:**
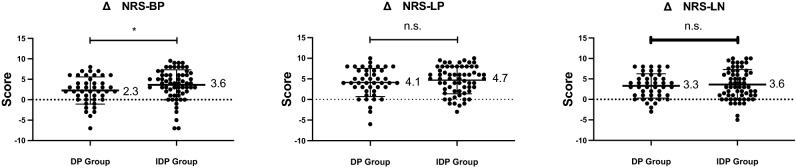
Graphs showing improved NRS scores (DNRS, calculated as preoperative NRS—postoperative NRS) in the two groups. n.s., not significant; *p < 0.05 indicates significant differences between groups. NRS, numeric rating scale; NRS-BP, NRS for low back pain; NRS-LP, NRS for leg pain; NRS-LN, NRS for leg numbness.

The improvements in each NRS score from before to after surgery were calculated. The change in NRS-BP score (ΔNRS-BP) differed significantly between the two groups: 3.6 ± 3.7 and 2.3 ± 3.3 in the DP and IDP groups, respectively (*p* < 0.05). However, the ΔNRS-LP and ΔNRS-LN scores did not differ significantly between the two groups (Fig. 3). We also analyzed the correlations between the PDQ-J and each NRS scores, including the NRS-BP, NRS-LP, and NRS-LN scores. Preoperative and postoperative NRS scores correlated weakly or moderately with the PDQ-J score (r = 0.285 to 0.496), but the DNRS score did not correlate significantly with the PDQ-J score component (Table 3).

**Table 3:** Correlation coefficient analysis of PDQ-J and each NRS scores.

	PDQ-J	Preoperative NRS BP	Preoperative NRS LP	Preoperative NRS LN
PDQ-J	1.000	0.376***	0.285**	0.496***
Preoperative NRS BP	0.377***	1.000	0.420***	0.490***
Preoperative NRS LP	0.285**	0.420***	1.000	0.590***
Preoperative NRS LN	0.496***	0.490***	0.590***	1.000

	PDQ-J	Postoperative NRS BP	Postoperative NRS LP	Postoperative NRS LN
PDQ-J	1.000	0.360***	0.375***	0.360***
Postoperative NRS BP	0.360***	1.000	0.739***	0.594***
Postoperative NRS LP	0.375***	0.739***	1.000	0.690***
Postoperative NRS LN	0.360***	0.594***	0.690***	1.000

	PDQ-J	ΔNRS BP	ΔNRS LP	ΔNRS LN
PDQ-J	1.000	− 0.025	− 0.164	0.093
ΔNRS BP	− 0.025	1.000	0.533***	0.492***
ΔNRS LP	− 0.164	0.533***	1.000	0.587***
ΔNRS LN	0.093	0.492***	0.587***	1.000

NRS, Numeric Rating Scale; NRS-BP, Numeric Rating Scale for low back pain; NRS-LP, Numeric Rating Scale for leg pain; NRS-LN, Numeric Rating Scale for leg numbness.

*p < 0.01, ***p < 0.001 indicates significant differences between groups.

Investigation of any complications showed that 11 patients in the IDP group had transient thigh pain and motor weakness, but they all recovered within three months after the operation. Transient neurological complications were the most common and accounted for 17.7% of the complications. By contrast, the perioperative complications in the DP group included incidental durotomy in five patients and neurological deterioration in three patients. One patient was due to hematoma of the neurological deterioration, and the other was due to implant failure. These two patients had improved symptoms after an early reoperation. Another patient considered the effects of the intraoperative disability. None of the patients exhibited deep infection or vascular or visceral injuries.

## Discussion

The treatment goal of MIS in patients with lumbar degenerative spinal conditions is to avoid or reduce tissue damage, and ultimately to improve preoperative pain. The use of MIS approaches has increased in recent years because LLIF and MIS–TLIF are associated with reduced intraoperative blood loss and infection rates, and improved postoperative pain and quality of life compared with conventional posterior fusion^11^.

However, few studies have compared LLIF with the other procedures, and further studies are needed to compare the results of LLIF with those of traditional lumbar interbody fusion approaches. Sembrano et al. compared ALIF, LLIF, TLIF, and posterior spinal fusion in 6- week radiographic outcomes. They concluded that LLIF could improve sagittal contour and other interbody fusion approaches and is superior to posterior fusion in disc height restoration^12^. A study by Kono et al. of 40 patients was compared by surgery type; mini-open TLIF (20 patients) and XLIF (20 patients), with a follow up of 1 year in terms of clinical results and radiological change. Postoperative clinical results were equally favorable for both procedures. However, XLIF had significantly improved correction of disc height compared to the mini-open TLIF^13^.

Significant improvement was noted in clinical outcomes including VAS. In a previous prospective analysis of postoperative pain progression in 600 patients treated with LLIF for degenerative spinal conditions, Rodgers et al. showed that the average VAS score decreased from 8.8 to 3.1 after LLIF, immediate improvement of 65%^14^. Phillips et al. also evaluated 82 of 107 patients with degenerative scoliosis who were available for the 24-month follow-up and found that the VAS scores for back and leg improved significantly from before the operation to the 24-month follow-up. The mean VAS score was reduced by half within five months after the surgery and remained constant thereafter. The mean improvement in VAS scores from before to 24 months after the operation were 3.4 for back pain and 3.5 for leg pain^15^. Based on this background, our study compared the clinical results of patients who received DP or IDP. We focused on the NRS scores. The patients who underwent IDP surgery showed significantly higher low back pain, leg pain, and leg numbness scores at the final follow-up compared with those who underwent DP surgery, although no differences were found about 12 months after surgery. However, a comparison of the improvement in NRS scores between the two groups showed that the IDP group exhibited more significant improvement in low back pain, possibly because invasive surgery was not performed, which would have limited the stress on the posterior support elements such as the facet joints, lamina, and paraspinal muscles.

On the other hand, despite its overall success, LLIF has been reported to have some unique complications, such as hip flexion deficit and lumbar plexus related motor deficits. Tomeh et al. reported that 27.5% of 102 patients who underwent LLIF experienced postoperative hip flexion weakness, which was transient and typically resolved within the first two weeks after surgery^16^. Salzmann et al.^17^ also reported rates of early postoperative anterior thigh pain and sensory and motor deficits in a cohort of 451 patients who underwent LLIF were 38.5%, 38.0%, and 23.9%, respectively. The authors also found that their percentage have decreased over time. According to a systemic review that compared the complications between MIS–TLIF and LLIF, both surgeries have acceptable complication profiles, although LLIF has an overall significantly higher complication rate than MIS–TLIF (31.4% vs. 19.2%). For specific complications, LLIF has higher rates of sensory as well as temporary and permanent neurological symptoms but does not have a significantly lower risk of intraoperative complications (1.93% for LLIF vs. 3.57% for MIS–TLIF)^11^. In our study, transient neurological complications such as hip flexion weakness, anterior thigh pain, and sensory and motor deficits were the most common and accounted for 17.7% of all complications. This percentage is similar to that reported previously. There have been several reports of the causes of these symptoms^16,18,19^. We believe that preventing blind dissection of the psoas muscle and minimizing the operation time required for LLIF may be effective for preventing these neurological complications^10^.

The present study has limitations such as its retrospective design, small sample size, short follow-up, and lack of multiple-factor analysis. The longer-term clinical data are necessary for determining pseudarthrosis rates. We also think that the surgical indications for some degenerative diseases should be strictly defined in future prospective studies. The L5/S1 level is not accessible for an LLIF, and the levels at L3/4 and above are typically better treated by an LLIF. Therefore, evaluation is the most important at the L4/5 level. If the number of patients increases, we would like to investigate procedures using only at the L4/5 level. Further prospective randomized clinical trials are needed to investigate whether MIS surgery is advantageous for improving postoperative pain.

## Conclusions

To our knowledge, this is the first study to focus on the changes in low back pain, leg pain, and leg numbness from before to after DP (MIS–TLIF) and IDP (XLIF) surgery to treat patients with DS with stenosis. The severe complications' rate might be inferior in the IDP compared with the DP surgery.

The estimated blood loss was lower, and the operation time was shorter in the IDP than in the DP group. Both groups exhibited improved low back pain, leg pain, and leg numbness, but the IDP group had a more significant improvement in low back pain than the DP group. Differences in the invasiveness of the procedures involving the posterior support elements, such as the facet joints, lamina, and paraspinal muscles, may be responsible for the differences between groups. However, more studies with longer follow-up are necessary to evaluate the IDP approach's theoretical benefit and assess whether the results of IDP are superior and durable as the ones achieved by the DP approach.

## Material and methods

This study was approved by the Institutional Review Board of Tokai University School of Medicine, the House Clinical Study Committee, and the Profit Reciprocity Committee, and all of the methods were carried out in accordance with relevant guidelines and regulations. Because this study was retrospective, informed consent was not obtained from each patient and the requirement for informed consent was waived (IRB Approval No.: 19R-277).

### Patients

We conducted this retrospective single-center study by reviewing the surgical and clinical data of patients diagnosed with degenerative spondylolisthesis (DS) with restenosis who underwent lumbar decompression and surgical intervertebral fusion.

The inclusion criteria were age > 18 years, lumbar spinal canal stenosis and lumbar DS, and undergoing a combined operation (IDP) using LLIF or MIS–TLIF at a single institute from January 2016 to October 2019. We started performing IDP using LLIF in this institution in 2016; before that, only MIS–TLIF was performed.

All patients were diagnosed based on a detailed history, neurological examination, radiographic examination, myelography, computed tomography after myelography, and/or magnetic resonance imaging (MRI). The main indication was neurogenic claudication caused by central or foraminal spinal stenosis. The conditions for a diagnosis of spondylolisthesis and the inclusion criteria for fusion surgery were > 5% slip of the lumbar vertebra in a neutral position or > 3-mm translation between flexion and extension positions radiographic evaluation. The operating surgeons recorded the location of stenosis based on their evaluation of the preoperative imaging studies. The exclusion criteria included previous lumbar spinal surgery at the same level or receipt of combined procedures, including direct posterior decompression and posterior lumbar fusion^20^.

The patients were categorized into two groups based on the operative procedure: the DP and IDP groups. Data were collected retrospectively and included demographics, operative details, the preoperative Japanese version of the painDETECT questionnaire (PDQ-J) score, numerical rating scale (NRS) scores, and postoperative neurological complications.

### Surgical technique

Patients in the DP group who received the MIS–TLIF were placed in the prone position. The facet joints and the ligamentum flavum were removed using a laminectomy rongeur, and the traversing nerve roots, exiting nerve roots, and lateral edge of the dura were exposed and decompressed. The annulus was incised, and a complete discectomy was performed. After confirmation of the spacer size, a polyether ether ketone cage filled with autologous cancellous bone was inserted accurately. The percutaneous pedicle screws (PPSs) were placed after the decompression. A bent rod was placed to connect the PPSs, and the intervertebral space was moderately compressed to ensure that the cage was very solid.

All patients in the IDP group who received XLIF and PPS fixation underwent MIS–LLIF surgery performed using the XLIF technique. The XLIF procedure has been described previously^20,21^. After the LLIF procedure, the patient was repositioned in the prone position (dual position) or the lateral decubitus position (single position), and the PPS was inserted. Posterior lumbar fixation using PPSs was then performed using fluoroscopy or an O-arm-based navigation system. The reference frame placed at the cranial spinous process was used for navigation.

### Evaluation

The PDQ established by Freynhagen et al.^22^ was used to identify the presence of neuropathic pain. The final score ranges from − 1 to 38 and indicates the likelihood of a neuropathic component. A score of ≤ 12 indicates a low likelihood of a neuropathic component, and a score of 19 suggests a high likelihood of a neuropathic component. The cutoff values for categorizing the type of pain based on the PDQ neuropathic pain-screening questionnaire scores were as follows: nociceptive pain, 0–12; unclear type of pain (mixed nociceptive and neuropathic pain), 13–18; and neuropathic pain, 19–38. The PDQ-J has been validated and has good reliability and validity, according to a study by Matsubayashi et al.^23^.

NRS scores were recorded using a 0–10 scale with 0 = no pain or numbness and 10 = the worst pain imaginable or numbness and were used to evaluate low back pain (NRS-BP), leg pain (NRS-LP), and leg numbness (NRS-LN). For all participants who were followed, data were obtained preoperatively and at the time of the final follow-up to complete the clinical evaluation.

### Statistical analysis

All data are expressed as mean ± standard deviation. Univariate differences between DP and IDP groups were assessed using independent-sample t-tests or the Mann–Whitney U test for data that were not normally distributed. The correlations between the PDQ-J and NRS scores were analyzed with the spearman’s product-moment correlation coefficient. All statistical analyses were performed with SPSS version 20.0 (IBM Corp., Armonk, NY, USA), and a *p *value < 0.05 was considered statistically significant^20,21^.
